# The Pseudokinase NIPI-4 Is a Novel Regulator of Antimicrobial Peptide Gene Expression

**DOI:** 10.1371/journal.pone.0033887

**Published:** 2012-03-21

**Authors:** Sid ahmed Labed, Shizue Omi, Martha Gut, Jonathan J. Ewbank, Nathalie Pujol

**Affiliations:** 1 Centre d'Immunologie de Marseille-Luminy (CIML), Aix-Marseille University, UM2, Marseille, France; 2 Institut National de la Santé et de la Recherche Médicale (INSERM), U1104, Marseille, France; 3 Centre National de la Recherche Scientifique (CNRS), UMR7280, Marseille, France; 4 Centre Nacional d'Anàlisi Genòmica, Barcelona, Spain; French National Centre for Scientific Research - Université Aix-Marseille, France

## Abstract

Hosts have developed diverse mechanisms to counter the pathogens they face in their natural environment. Throughout the plant and animal kingdoms, the up-regulation of antimicrobial peptides is a common response to infection. In *C. elegans*, infection with the natural pathogen *Drechmeria coniospora* leads to rapid induction of antimicrobial peptide gene expression in the epidermis. Through a large genetic screen we have isolated many new mutants that are incapable of upregulating the antimicrobial peptide *nlp-29* in response to infection (i.e. with a Nipi or ‘no induction of peptide after infection’ phenotype). More than half of the newly isolated Nipi mutants do not correspond to genes previously associated with the regulation of antimicrobial peptides. One of these, *nipi-4*, encodes a member of a nematode-specific kinase family. NIPI-4 is predicted to be catalytically inactive, thus to be a pseudokinase. It acts in the epidermis downstream of the PKC∂ TPA-1, as a positive regulator of *nlp* antimicrobial peptide gene expression after infection. It also controls the constitutive expression of antimicrobial peptide genes of the *cnc* family that are targets of TGFß regulation. Our results open the way for a more detailed understanding of how host defense pathways can be molded by environmental pathogens.

## Introduction

Pathogenic microorganisms represent one of the most ubiquitous and powerful sources of selection for higher eukaryotes including humans [1]. Different pathogens have specific natural host tropisms, sometimes broad, as in the case of *Pseudomonas aeruginosa* [2], [3], and in other cases, such as HIV, very narrow. Part of this tropism reflects the divergent mechanisms of host resistance, as exemplified by cultivar-specific resistance in plants [4]. The evolution of adaptive immunity is often cited as an extreme example of immune system evolution. But even among invertebrates that rely on their innate immune systems, there is evidence for considerable variation from the phylum to the species level. For example, in contrast to most other animal species, nematodes, including *Caenorhabditis elegans*, have lost NF-κB, a key transcription factor in immunity [5], [6], [7]. Studying the interaction of *C. elegans* with its natural pathogens therefore sheds light on NF-κB-independent defense pathways.

A number of natural pathogens of *C. elegans* have been identified, including viruses [8], microsporidia [9], and bacteria such as *Microbacterium nematophilum* [10] and *Serratia marcescens* [11], [12]. *Drechmeria coniospora* is a nematophagous fungus that infects *C. elegans* and other species of nematodes [13]. When *C. elegans* is sampled from its natural environment, it is often found to be infected with *D. coniospora* (M-A. Felix, personal communication). *D. coniospora* produces adhesive conidia that attach to the worm's cuticle. These germinate to produce invasive hyphae that penetrate the cuticle and grow throughout the epidermis [14]. In *C. elegans*, infection with *D. coniospora* provokes an innate immune response in the epidermis involving the expression of a large number of genes including those encoding antimicrobial peptides (AMPs) of the NLP and CNC families [15], [16], [17].

Certain members of each family are found in 2 distinct genomic groups, comprising *nlp*-*27*, *28*, *29*, *30*, *31* and *34*, referred to as the *nlp-29* cluster, and *cnc*-*1*, *2*, *3*, *4*, *5* and *11*, the *cnc*-*2* cluster. The induction of expression of the genes of the *nlp-29* cluster is strongly dependent on the p38 MAPK *pmk-1*, while that of the *cnc*-2 cluster genes requires the TGFß *dbl-1*. The expression of all the genes of the *nlp-29* cluster, and some of those of the *cnc*-*2* cluster is also strongly increased in the epidermis if worms are physically injured. In this case, the up-regulation of both the *nlp* genes and *cnc*-*1*, *cnc*-*5* and *cnc*-*11* (but not *cnc*-*2* or *cnc*-*4*) is largely dependent upon *pmk-1* [16], [18], [19].

We have shown that for *nlp-29* cluster genes, following both infection and injury, inductive signaling passes via TPA-1, a protein kinase C delta (PKC∂) that acts upstream of TIR-1, the nematode ortholog of SARM, and a MAPK cassette comprising the MAP3K NSY-1, the MAP2K SEK-1, and PMK-1 [20]. This cascade acts upstream of the STAT-like transcription factor STA-2 that physically interacts with the C-terminus of the SLC6 transporter SNF-12 [21]. SNF-12 is found in endosome-like vesicles in the epidermis, where it may act as a signaling platform during the innate immune response. The elements that contribute to signaling upstream of TPA-1/PKC∂ have only been partially characterized. Wounding and infection require G-protein signaling, involving the Gα protein GPA-12 and the Gß RACK-1, while infection specifically involves the Tribbles-like kinase NIPI-3 [18], [20].

In addition to provoking the increased expression of AMPs, wounding also triggers a rise in intracellular Ca^2+^. This is controlled by an epidermal signal transduction pathway that includes the Gα(q) EGL-30. This pathway is required for actin-dependent wound closure, but not for injury-induced AMP expression [22]. On the other hand, the Death-associated protein kinase DAPK-1 negatively regulates wound repair and AMP gene expression [23]. Many, but not all, of the elements that act in the epidermis also mediate the innate defenses against intestinal pathogens and toxins [24], [25], [26], [27], [28], [29], [30], [31], [32]. Conversely, certain genes that participate in p38 MAPK signaling in the intestine, including *dfk-2* [28] are not required for the induction of *nlp-29* [20].

Our current understanding of both epidermal and intestinal innate immunity is far from complete. In the current study, we therefore undertook a large genetic screen for components of the signaling pathways that control AMP gene expression in the epidermis. We isolated and mapped 26 mutant alleles, uncovering 6 new genes required for AMP gene induction after *D. coniospora* infection. We cloned one of these genes, *nipi-4* (*nipi* for “no induction of peptide after *Drechmeria* infection”). We show here that *nipi-4* encodes a nematode-specific protein with a kinase-like domain that is predicted to be a pseudokinase. It acts downstream of PKC∂/TPA-1, which was previously shown to modulate the activity of a conserved p38 MAPK cassette [20]. This provides an illustration of an animal family-specific modulation of an innate immune signaling pathway.

## Results

### A genetic screen for Nipi mutants

**Figure 1:**
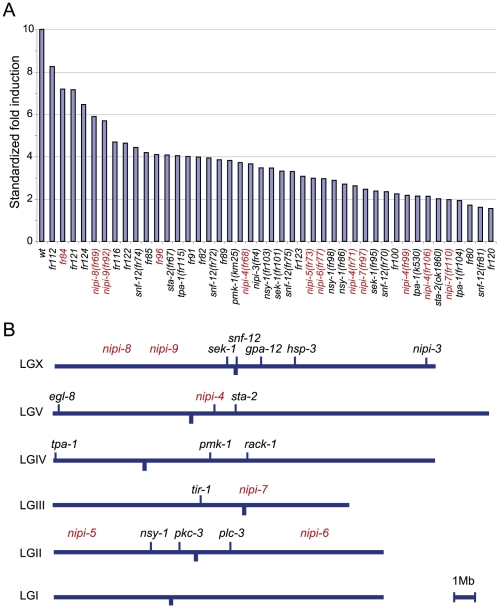
New Nipi alleles isolated in a large scale screen. (A) Biosort quantification of the fluorescence in wild type and different mutants strains carrying an integrated P*nlp-29*::GFP reporter (*frIs7*) following infection including *sta-2(ok1860)*, *nipi-3(fr4)*, *tpa-1(k530)* and 38 new alleles, 11 of which have been determined to define 6 new independent complementation groups. The average fold induction for each strain is represented after standardization across different independent experiments by normalizing to 10 the fold induction between the wild type strain infected versus non infected. (B) Genetic map of Nipi loci identified from screens or from candidate gene approaches. The map has been scaled to the genome sequence, as in [48]. The Nipi genes identified in the present mutagenesis and in previous studies [15], [18], [20], [21] (Couillault *et al.* submitted) are represented in red and black respectively.

We undertook a large-scale genetic screen for mutants that prevented the normal induction of a P*nlp-29*::GFP reporter transgene after infection with *D. coniospora*. From 130,000 mutagenized haploid genomes, we isolated 57 candidate mutant strains. These were then subjected to a confirmatory round of screening and outcrossing. We retained 44 mutant strains that had a sufficiently penetrant phenotype. All behaved as if they were carrying simple recessive alleles. To characterize these in further detail, we first quantified reporter gene expression in uninfected and infected worms. All mutants showed a reduction of P*nlp-29*::GFP induction with the most penetrant alleles showing essentially a complete block of the reporter (Figure 1A).

Three strains exhibited resistance to the phorbol ester PMA. As the only gene known to provoke PMA-resistance in *C. elegans* is *tpa-1* [33], [34], we sequenced this gene in one mutant and thus identified a G384E mutation. We presume that the other two mutants are also *tpa-1* alleles, but did not characterize them as there are already more than 50 available *tpa-1* alleles. For the other 41 strains, we performed classical SNP mapping to assign alleles to individual chromosomes, which was unambiguous for 26 of them. We then performed targeted complementation tests, between alleles, and with candidate genes on the appropriate chromosome. When a new allele failed to complement a candidate gene, the corresponding gene from the mutant was sequenced. This allowed the identification of new alleles for 4 previously characterized Nipi genes, 6 for *snf-12*, 3 for *nsy-1*, 2 for *sek-1* and 1 for *sta-2*. These numbers give an indication of the degree of saturation of the screen. The remaining alleles appear to correspond to previously uncharacterized genes. They fall into 6 complementation groups, some represented by multiple alleles (Figure 1B, Table 1).

**Table 1:** New Nipi alleles.

Allele	Genomic position^1^	Gene	Mutation	Protein modification
*fr103*	II:5,023,842	*nsy-1*	C to T	nonsense
*fr98*	II:5,026,498	*nsy-1*	C to T	nonsense
*fr86*	II:5,026,945	*nsy-1*	G to A	E836K
*fr76*	IV	*tpa-1*	Unknown	Unknown
*fr115*	IV	*tpa-1*	Unknown	Unknown
*fr104*	IV:105,994	*tpa-1*	G to A	G384E (B0545.1a)
*fr71*	V:7,869,395	*nipi-4*	C to T	nonsense
*fr68*	V:7,869,401	*nipi-4*	G to A	G313E
*fr99*	V:7,869,753	*nipi-4*	G to A	Splice acceptor
*fr106*	V:7,870,474	*nipi-4*	C to T	nonsense
*fr67*	V:9,741,675	*sta-2*	G to A	nonsense
*fr101*	X:7,818,617	*sek-1*	G to A	D212N
*fr95*	X:7,818,822	*sek-1*	G to A	G194R
*fr75*	X:9,030,736	*snf-12*	G to A	nonsense
*fr72*	X:9,031,202	*snf-12*	G to A	G391R
*fr74*	X:9,031,495	*snf-12*	G to A	E472K
*fr81*	X:9,032,001	*snf-12*	T to G	nonsense
*fr102*	X:9,032,231	*snf-12*	TA insertion	Frame shift
*fr70*	X:9,032,686	*snf-12*	G to A	R792K
*fr73*	II	*nipi-5*		
*fr77*	II	*nipi-6*		
*fr97*	III	*nipi-7*		
*fr108*	III	*nipi-7*		
*fr110*	III	*nipi-7*		
*fr69*	X	*nipi-8*		
*fr92*	X	*nipi-9*		

1 WormBase Release WS228.

### Molecular identification of *nipi-4*

**Figure 2:**
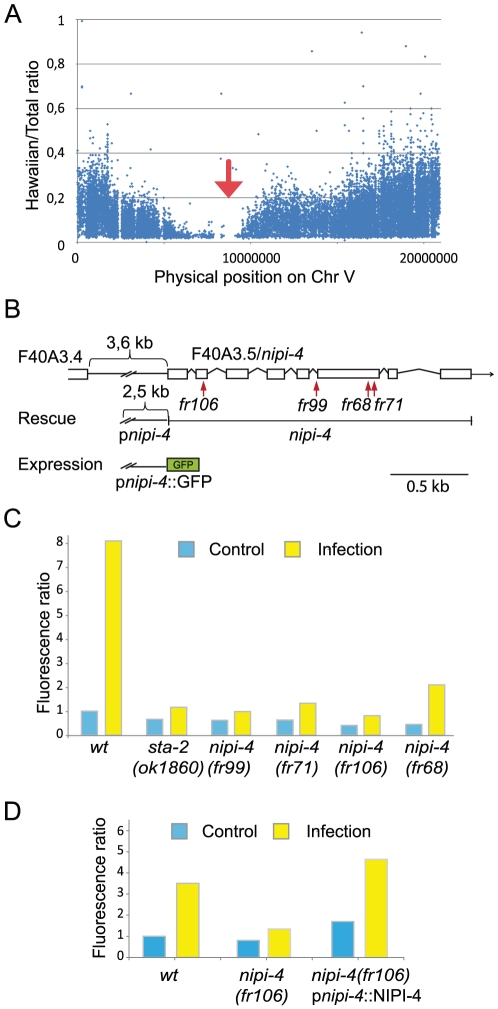
*nipi-4* encodes a pseudokinase required for the induction of *nlp-29*. (A) SNP mapping with WGS. The positions of SNP loci on Chromosome V for the *fr106* allele are depicted as a XY scatter plot, where the ratio ‘Hawaiian/total number of reads’ for each SNP is represented, as in [35]. The region without Hawaiian SNPs contains the mutation (red arrow). (B) Exon-intron structure of *nipi-4*, adapted from WormBase (WS220), with the positions of the *fr68*, *fr71*, *fr99* and *fr106* mutations indicated. Also shown is the structure of the p*nipi-4*::GFP & p*nipi-4*::NIPI-4 constructs. (C) Biosort quantification of the normalized fluorescence ratio in wild type, *sta-2(ok1860)* and the 4 *nipi-4* alleles *fr68*, *fr71*, *fr99* and *fr106* carrying *frIs7* following infection. For this and subsequent figures, see Materials and Methods for details of the data processing and the number of worms analyzed. The results are representative of 3 independent experiments. (D) Biosort quantification of the normalized fluorescence ratio in wild type, *nipi-4(fr106)* and *nipi-4(fr106)* with a rescuing transgene p*nipi-4*::NIPI-4, carrying *frIs7* following infection.

One complementation group was given the name *nipi-4* and characterized in detail. Whole-genome resequencing of pooled recombinants [35] between *nipi-4*(*fr106*) and the polymorphic Hawaiian strain CB4856 clearly delineated a candidate region for the mutation on the center of chromosome V (Figure 2A). Within this region, only one nonsense mutation was found, in the gene F40A3.5, which is predicted to encode a 396 amino acid membrane-bound protein with a tyrosine kinase domain [36] (Figure S1). Sequencing this gene from the other *nipi-4* alleles revealed 3 independent mutations, a different nonsense mutation in *fr71*, an alteration of a splice acceptor site in *fr99* that would be predicted to lead to a severely truncated protein, and a missense mutation in the kinase domain in *fr68* (Figure 2B, Table 1). This very strongly suggests that *nipi-4* corresponds to F40A3.5. In contrast to *fr68*, which has a milder phenotype, the alleles *fr71*, *fr99* and *fr106* all provoke similarly penetrant phenotype and are predicted to correspond to null alleles (Figure 2C). Transformation rescue confirmed the identity of *nipi-4* as F40A3.5 (Figure 2D).

Interestingly, we have only identified NIPI-4/F40A3.5 orthologs in nematodes. The *Caenorhabditis* proteins, from *elegans*, *briggsae*, *brenneri*, *japonica* and *remanei* species, are predicted to be kinase-dead, since they lack the essential aspartic acid active site residue. In contrast, predicted NIPI-4 orthologs from non-*Caenorhabditis* species like *Ascaris suum* and *Pristionchus pacificus* are expected to be functional kinases. Conversely, only the *Caenorhabditis* proteins have a tyrosine in the predicted activation loop that could potentially be the target of phosphorylation (Figure S1). We discuss the significance of these observations below.

### *nipi-4* acts cell autonomously in epidermal cells

**Figure 3:**
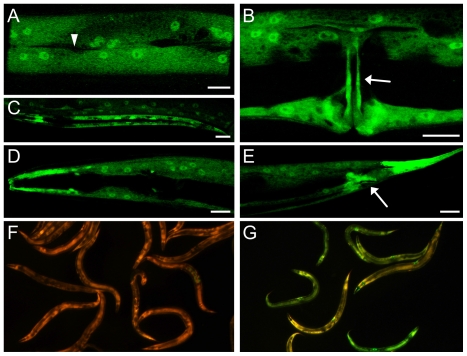
The *nipi-4* gene acts cell autonomously in the epidermis. (A–E) Expression of *nipi-4* is seen throughout the epidermis (A & B)), in larvae (C) and adults (A,B,D&E), from head (D) to tail (E), in vulval cells (arrow in B), in rectal cells (arrow in E), but not in the seam cells (arrowhead in A), scale bar 10 µm. (F–G) *nipi-4*(*fr71*) and *nipi-4*(*fr71*);*frEx496* (P*col-19*::NIPI-4) worms strains carrying an integrated P*nlp-29*::GFP reporter (*frIs7*) following infection. The expression of *nipi-4* in epidermal cells in the adult rescues the *nipi-4* phenotype. Green and red fluorescence is visualized simultaneously with a GFP long pass filter.

To identify the cells in which *nipi-4* is expressed, we generated transgenic animals carrying a GFP transcriptional reporter construct (Figure 2B). We observed expression in the epidermis of *C. elegans* throughout development (Figure 3A–E). This pattern overlaps with that of the previously characterized components of the PKC∂/p38 MAPK pathway, including *snf-12* and *sta-2* [21] and suggests that *nipi-4* may act in a cell-autonomous manner. To evaluate this directly, we generated transgenic animals in which the expression of *nipi-4* was under the control of the *col-19* promoter, which is expressed specifically in epidermal cells as animals enter adulthood [37]. In these worms we observed an essentially normal expression of P*nlp-29*::GFP upon infection (Figure 3F–G). On the other hand, expression of *nipi-4* in the intestine under the control of the *vha-6* promoter [38] did not give any rescue (Figure S2). Together, this indicates that *nipi-4* acts cell-autonomously in the epidermis to regulate antimicrobial peptide gene expression.

### *nipi-4* regulates AMP gene expression after infection and wounding

**Figure 4:**
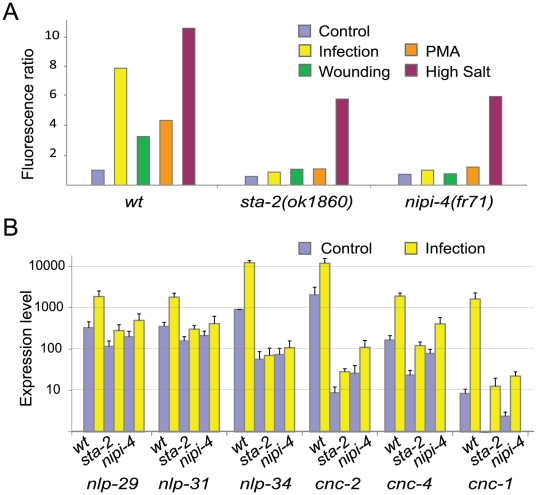
The *nipi-4* gene is required for the response to infection and wounding. (A) *nipi-4* mutants do not block the induction of *nlp-29* expression upon osmotic stress. Biosort quantification of the normalized fluorescence ratio in wild type, *sta-2(ok1860)* and *nipi-4(fr71)* worms carrying *frIs7* following infection by *D. coniospora*, wounding, PMA treatment and osmotic stress. (B) Quantitative RT-PCR analysis of gene expression levels in non- infected and infected wild type, *sta-2(ok1860)* and *nipi-4(fr106)* worms. The columns show the average expression level (arbitrary units) and SEM from 4 experiments. The level of *nlp-34* expression in control animals is set at 1024 (see Materials and Methods).

To define further the function of *nipi-4*, we assayed the expression of the P*nlp-29*::GFP reporter transgene in the *nipi-4* mutant background under other conditions that normally lead to its expression, including injury, exposure to PMA and osmotic stress [16], [18], [20]. In a *nipi-4(fr71)* mutant, in addition to a near-complete block of P*nlp-29*::GFP expression after infection, there was no induction of the reporter gene upon needle wounding or exposure to PMA. There was, however, a strong induction of P*nlp-29*::GFP expression upon exposure to high salt, comparable to that seen in a *sta-2* mutant. Similar results were obtained with *nipi-4(fr99)* and *nipi-4(fr106)* (Figure 4A, and results not shown). This suggests that *nipi-4* acts downstream of the PKC∂ TPA-1 to regulate *nlp-29* expression specifically after wounding and infection.

We also analyzed the expression of other genes that have been shown to be induced upon *D. coniospora* infection [16]. We could confirm by qRT-PCR that in the *nipi-4(fr106)* mutant, just as in *sta-2* or *snf-12* mutants [21], the induction of *nlp-29* after infection was essentially abrogated. Two other genes of the *nlp-29* cluster, *nlp-31* and *nlp-34* were similarly affected (Figure 4B). It is interesting to note that in the *nipi-4* and *sta-2* mutants the constitutive expression of *nlp-34* was reduced by 10 fold whereas it was not greatly changed for *nlp-29* and *nlp-31*. The genes of the *cnc-2* cluster are regulated in a manner distinct from *nlp-29* as their induction after *D. coniospora* infection is p38 MAPK independent. Rather their induction requires signaling via a non-canonical TGFβ/DBL-1 pathway [19]. We found by qRT-PCR that loss of function of *nipi-4* strongly affected the constitutive expression of *cnc-1* and *cnc-2* and to a lesser extent *cnc-4*. This parallels the phenotype due to loss of *sta-2* function (Figure 4B), as well as *snf-12* [21]. Indeed, as discussed below, the constitutive expression of these genes was reduced to such a degree that it is technically difficult to evaluate the extent of gene induction after infection. Thus, like *snf-12* and *sta-2*, *nipi-4* plays a role in innate immune signaling and influences targets of both the PKC∂/p38 MAPK/PMK-1 and TGFβ/DBL-1 pathways.

### Modulation of AMP gene expression by *gpa-12* requires *nipi-4*

**Figure 5:**
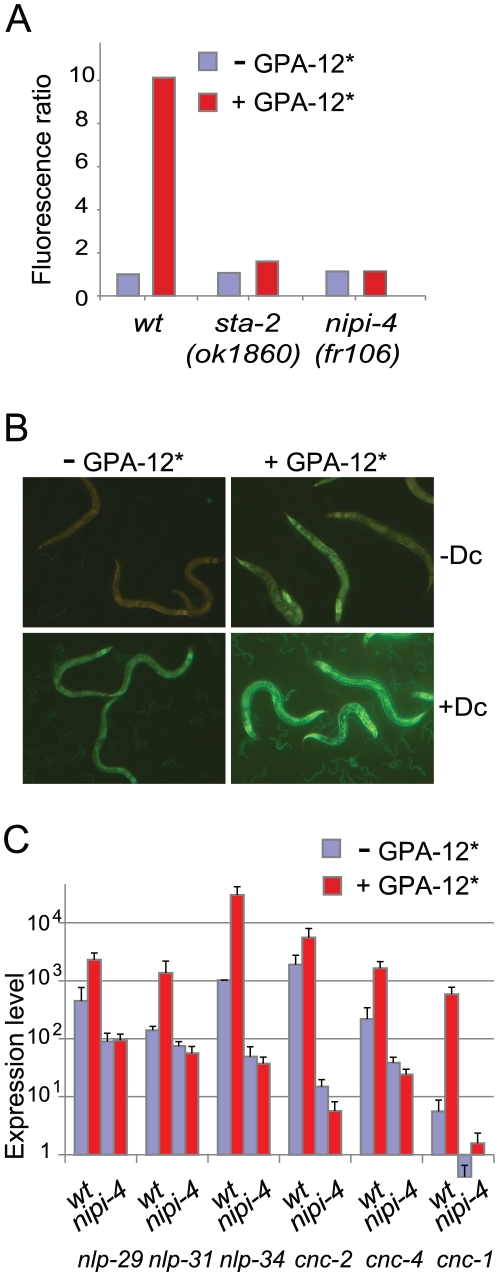
*nipi-4* genetically interacts with the G-protein/PKCδ/p38 MAPK cascade. (A) The G-protein/PKCδ/p38 MAPK cascade regulates the expression of *nlp-29* after infection and wounding. Biosort quantification of the normalized fluorescence ratio in wild type, *sta-2(ok1860)* and *nipi-4(fr106)* mutant worms carrying an integrated P*nlp-29*::GFP reporter, with or without a transgene carrying an activated form of GPA-12 under the control of an epidermis promoter (P*col-19*::GPA-12*). (B) Images of the wild type strain carrying *frIs7* with (+GPA-12*) or without (−GPA-12*) P*col-19*::GPA-12* in control animal (−Dc) or worm infected by *D. coniospora* (+Dc). Green and red fluorescence is visualized simultaneously. (C) Quantitative RT-PCR analysis of gene expression levels in wild type and *nipi-4(fr106)* worms with or without P*col-19*::GPA-12*. The columns show the average expression level (arbitrary units) and SEM from 3 experiments. The level of *nlp-34* expression in control animals is set at 1024 (see Materials and Methods).

Our previous dissection of the innate immune signaling pathways that govern AMP expression in the epidermis relied on the use of PMA to activate TPA-1/PKC∂ and an active form of the Gα protein GPA-12 (GPA-12* [34]), produced under the control of a heat-shock promoter [20], [21]. As both PMA and heat-shock have pleiotropic effects on the physiology of *C. elegans*, we developed a more refined tool, with GPA-12* under the control of the *col-19* promoter, driving its expression in the adult epidermis [37]. We injected this construct into worms carrying an integrated P*nlp-29*::GFP reporter. In uninfected transgenic worms carrying the P*col-19*::GPA-12* construct, we observed a very marked increase in the expression of P*nlp-29*::GFP in the epidermis from the late L4 stage onwards. The level of reporter gene expression was even further increased upon infection with *D. coniospora* (Figure 5A & B). As expected, increased P*nlp-29*::GFP expression was totally abrogated in a *tpa-1* mutant (results not shown). We found by qRT-PCR that the transgenic strain exhibited an elevated constitutive expression of *nlp-29*, *nlp-31* and *nlp-34*, and *cnc-1*, *cnc-4*, and to a lesser extent *cnc-2* (Figure 5C). The results mirrored to a striking degree the pattern of gene expression changes induced by infection (Figure 4B). When we crossed the P*col-19*::GPA-12* transgene into *nipi-4(fr106)* mutant, the elevated expression of the P*nlp-29*::GFP reporter provoked by the active form of GPA-12 was abrogated, to a similar degree as in the *sta-2* mutant background (Figure 5A). The effect of GPA-12* on the expression of other *cnc* and *nlp* genes was also abolished in the *nipi-4(fr106)* mutant, as judged by qRT-PCR (Figure 5C). Together, these results confirm the role of *nipi-4* as a novel regulator of AMP gene expression during the infection of the worm, acting genetically downstream of PKC∂ TPA-1.

## Discussion

To characterize the molecular pathways that underpin anti-fungal innate immunity in *C. elegans*, we previously undertook a small-scale genetic screen for genes required for the induction of an AMP reporter gene after infection. We isolated and characterized 5 alleles that fall into 4 complementation groups. This provided a framework to understand the regulation of antimicrobial peptide expression in the *C. elegans* epidermis [39]. In the current study, we chose to extend the approach, with the aim of performing a saturating screen. Counting just the alleles that were amenable to classical SNP mapping, we found a total of 11 complementation groups, of which 2 corresponded to genes that had been hit in the previous screen, and 3 to genes previously known to be involved in the regulation of *nlp* genes, leaving a total of 6 new complementation groups.

The current genetic screen has reinforced the importance of the p38 MAPK pathway as a central part of anti-fungal defenses in the epidermis as we recovered multiple alleles of the MAP3K *nsy-1* and the MAP2K *sek-1*. A common p38 MAPK signaling cassette is also required for resistance to intestinal bacterial infection. In a screen for genes that both regulate the constitutive expression of the intestinal gene T24B8.5 and that are required for resistance to *P. aeruginosa* infection, 14 *nsy-1*, 8 *sek-1*, 7 *pmk-1* and 3 *tir-1* alleles were isolated, together with one allele of a new actor in the intestinal p38 pathway, the transcription factor *atf-7* [29]. Our screens might have allowed the identification of many more distinct genes, presumably because they were less stringent. We may well find alleles of *pmk-1* and *tir-1* among the alleles that have yet to be mapped.

The genes of the *cnc-2* and *nlp-29* clusters share a common evolutionary origin [16]. It is reasonable to imagine that they were initially regulated by a common mechanism, but this is clearly no longer the case. Certain genes, however, participate in the regulation of both groups of genes. Indeed, the results we obtained using *nipi-4* have reinforced observations that we previously made with *snf-12* and *sta-2* mutants [21]. These 3 genes are necessary for the induction of the genes of the *nlp-29* cluster, but do not greatly affect their constitutive level of expression. On the other hand, loss of function of any one of the 3 essentially abolishes the constitutive expression of *cnc-1* and *cnc-2*. The drop in the constitutive level of its expression is such that it is not technically possible for us to determine reliably by qRT-PCR whether *D. coniospora* infection still provokes an induction of these genes in the *nipi-4*, *snf-12* or *sta-2* mutants. In wild-type worms, the induction of the *cnc* genes is almost entirely dependent upon *dbl-1*. Changes in *dbl-1* expression do not however affect their constitutive expression. Nor does *dbl-1* have any effect on the induction of the genes of the *nlp-29* cluster [19]. This suggests a model in which *nipi-4*, *snf-12* and *sta-2* are involved in two different processes, one that governs the constitutive expression of *cnc-1* and *cnc-2*, and the other that controls the inducibility of genes of the *nlp-29* cluster. The degree to which these two functions are interdependent remains to be established, as does the exact impact of *nipi-4* on *cnc-4*, as it is less clear-cut.

In addition to these open questions, the structure of NIPI-4 itself raises a number of issues. As mentioned above, NIPI-4-like proteins are only found in a subset of nematodes, and are never duplicated. Certain defense mechanisms have been lost in parasitic nematodes such as *Meloidogyne incognita* [40], but a NIPI-4 homolog can be found in *Meloidogyne* species and in the animal parasite *Ascaris suum*, so this protein is not restricted to free-living nematodes. Interestingly, while orthologs in *A. suum* and *Pristionchus pacificus* possess the characteristic catalytic aspartate residue, this residue is absent in all *Caenorhabditis* species, so these proteins are predicted to be catalytically inactive pseudokinases. This suggests that NIPI-4 has evolved a kinase-independent function in *Caenorhabditis* species. NIPI-4 might compete with one or more active kinases for substrates or binding partners. In such a scenario, the putative kinase(s) would need to play a negative regulatory role. The loss of catalytic activity in NIPI-4 has, however, been mirrored by the acquisition of a potential activation loop phosphorylation site not seen in other species. It is therefore tempting to speculate that NIPI-4 in *Caenorhabditis* species is able to donate its activation loop to another kinase following heterodimerization, as is seen for example with STRAD and LKB1 [41]. NIPI-4 and all its identified orthologs possess a predicted transmembrane segment, N-terminal to the kinase domain that could allow its association with the membrane of one or more classes of intracellular vesicles. We previously showed that SNF-12 is found in endosome-like vesicles, and that endocytosis is indispensable for the transcriptional response to infection [21]. One could conjecture that NIPI-4, SNF-12 and STA-2 form a signaling complex on endosomes that is activated following physical association with the MAPK PMK-1. Understanding the function of NIPI-4 at the biochemical and cellular level, an objective of future studies, will give insights into how a species-specific host defense pathway can be molded by the natural pathogens found in a particular environmental niche.

## Materials and Methods

### Nematode strains

All strains were maintained on nematode growth media (NGM) and fed with *E. coli* strain OP50, as described [42]. In addition to the wild-type strain N2 and CB4856 that were obtained from the *Caenorhabditis* Genetics Center (CGC), the following mutants were used for complementation tests all carrying the *frIs7* transgene containing the P*nlp-29*::GFP and P*col-12*::DsRed reporters [18] : *snf-12(tm692) X, sek-1(km4) X, hsp-3(ok1083) X, nsy-1(age3) II, tir-1(tm3036) III, tpa-1(k530) IV, egl-8(n488) V* and *sta-2(ok1860) V*. The 4 *nipi-4* alleles were outcrossed twice with N2.

### Mutants Isolation

We mutagenized IG274 wild type worms carrying the *frIs7* transgene with EMS using standard procedures [43]. 130,000 genomes were screened using the same criteria described in [18]. Briefly, synchronized F2 worms were infected at the L4 stage with *D. coniospora*. After 24 h at 25°C, we screened for worms that failed to show an elevated level of GFP expression after *D. coniospora* infection and transferred them onto nystatin containing NGM plates. Mutant alleles were mapped through standard genetic and bulk SNP mapping by analysis of 20 to 30 recombinants with the strain CB4856 [44]. Genetic complementation tests were done between mutants located on the same chromosome, defining 6 new independent complementation groups.

### Whole Genome Sequencing

*nipi-4(fr106)* mutation was further mapped and identified using a whole genome sequencing-SNP mapping protocol [35]. Briefly, *nipi-4(fr106)* was crossed with Hawaiian CB4856 males and 20 F2 mutant recombinant lines were isolated. The DNA of these pooled lines was prepared using a standard protocol with proteinase K lysis, RNAse A treatment and phenol/chloroform extraction. The pooled DNA was subjected to whole genome sequencing in multiplexed run with 4 samples in one sequencing lane of a v1.5 flowcell on HiSeq 2000 instrument, generating paired 100 nucleotide reads. The results were analyzed using Maqgene [45].

### Infection, wounding, exposure to high salt and PMA

Infections with *D. coniospora* and wounding were carried out at 25°C as described [18]. Briefly, animals were infected with *D. coniospora* at the L4 stage or exposed to high salt and incubated at 25°C. After 18 h, age-matched non-infected animals were used for wounding assays, exposure to PMA, or kept as control. Exposure of worms to high salt (350 mM NaCl) and PMA (1 µg/ml) were done on NGM plates as previously described [20].

### Constructs and transgenic lines

P*nipi-4*::GFP was obtained by Gateway cloning (Invitrogen™). A 2,521 bp fragment upstream of the *nipi-4* start site was amplified (with primers JEP1974-JEP1975), cloned into the pDONRP4-P1R vector, then transferred into the destination vector pDEST-DD04-Neo a generous gift from D. Dupuy [46] so that it was cloned upstream of the GFP::*unc-54*_3′UTR cassette. The P*nipi-4*::GFP was injected at 20 ng/µl together with P*ttx-3*::DsRed2 at 70 ng/µl into N2 worms. Two independent lines were generated showing the same expression pattern IG1341 *wt; frEx483* and IG1342 *wt; frEx484*.

P*nipi-4*::NIPI-4 (pMS18) was obtained by multisite recombinational Gateway cloning (Invitrogen™). A *nipi-4* genomic fragment comprising the entire ORF with the ATG but without the stop codon was amplified (JEP1964–JEP1965) and cloned into pDONR/Zeo (Invitrogen™). The promoter and gene entry clones were used together with a *unc-54*_3′UTR entry clone in a multi-partite LR reaction into the pJPDest R4R3 vector, to produce P*nipi-4*::NIPI-4. This construct was injected at 20 ng/µl together with pB*unc-53*::GFP [47] at 70 ng/µl into *nipi-4(fr106);frIs7*. One line was generated IG1343 *wt; frEx485*.

P*col-19*::NIPI-4 was obtained by Gateway cloning (Invitrogen™). The *nipi-4* gene entry clone described above was recombined into the destination vector pCZGY1434 that contains the promoter of *col-19* (P*col-19*), a generous gift from A. Chisholm [22]. This construct was injected at 30 ng/µl together with pB*unc-53*::GFP [47] at 70 ng/µl in IG1352 *nipi-4(fr71); frIs7*. Two lines were generated IG1404 *wt; frEx496* and IG1405 *wt; frEx497*.

P*vha-6*::NIPI-4::GFP (pMS21) was obtained by multisite recombinational Gateway cloning (Invitrogen™). A 1,255 bp genomic fragment upstream of the *vha-6* start site was amplified (with primers JEP1982–JEP1983), cloned into the pDONRP4-P1R vector. The *nipi-4* gene entry clone described above and the P*vha-6* promoter entry clone were used together with GFP entry clone in a multi-partite LR reaction into the pJPDest R4R3 vector. This construct was injected at 2 ng/µl together with pB*unc-53*::GFP [47] at 70 ng/µl in IG1352 *nipi-4(fr71); frIs7*. Two lines were generated IG1410 *wt; frEx498* and IG1411 *wt; frEx499*.

P*col-19*::GPA-12* was obtained by Gateway cloning (Invitrogen™). The DNA encoding an activated form of GPA-12 (with the Q205L mutation) was amplified from the construct pRP2205 a generous gift from R. Korswagen [34] with the primers JEP1976–JEP1977, inserted into a Gateway pDONR/Zeo (Invitrogen™) then recombined into the destination vector pCZGY1434 [22]. This construct was injected at 30 ng/µl together with pB*unc-53*::GFP [47] at 70 ng/µl in IG274 *wt; frIs7*. One line was generated IG1363 *wt; frEx486* and then subsequently integrated using Gamma rays and outcrossed several times with N2 generating IG1389 *wt; frIs7 IV; frIs30*.

### Analysis with the COPAS Biosort

Analysis of P*nlp-29*::GFP induction in the strain carrying the *frIs7* integrated array for the different treatments were all done at the same time on worms 24 h after the L4 stage with the COPAS Biosort (Union Biometrica™) [18]. The *frIs7* integrated array consists of two reporter transgenes, P*nlp-29*::GFP and P*col-12*::DsRed2. As the latter exhibits a constitutive expression in the epidermis that is unaffected by infection or other tested conditions, the fluorescence ratio green/red represents the variation in P*nlp-29*::GFP expression normalized for the size of individuals [18]. The mean values are shown normalized to the wild type control that is set to one. The number of animals used in each experiment is given below. As previously described [18], due to the nature of the distribution, standard deviations are not always an informative parameter when measuring fluorescent reporter gene expression using the Biosort. Data are, however, in all cases representative of at least 3 independent experiments.

### Number of animals quantified with the COPAS Biosort

Figure 2C: 231, 242, 197, 158, 91, 118, 139, 90, 122, 139, 67, 93

Figure 2D: 202, 182, 153, 123, 219, 120 (Combined 3 experiments)

Figure 4A: 104, 111, 98, 103, 158, 171, 160, 54, 72, 110, 139, 97, 85, 64, 81

Figure 5A: 286, 234, 542, 296, 515, 256 (Combined 3 experiments)

### qRT-PCR

L4 worms were infected for 6 h at 25°C with *D. coniospora*. 1 µg of total mRNA from infected and non-infected worms were used for reverse transcription (Applied Biosystems™). Quantitative real-time PCR were performed using 1 µl of cDNA in 10 µl of SYBERgreen Applied Biosystems™ and 0.1 µM of primers on a 7500 Fast Real-Time PCR System using *act-1* (JEP538-JEP539) as a control, with *nlp-29* (JEP848–JEP952), *nlp-31* (JEP950–JEP953), *nlp-34* (JEP969–JEP970), *cnc-1* (JEP1087–JEP1088), *cnc-2* (JEP944–JEP549) and *cnc-4* (JEP1124–JEP1125), for primer sequences see [21]. Results were normalized to *act-1*, and then relative expression calculated using 2((A+10)−x), A being the normalized cycle number for *nlp-34* in the non-infected sample and x the value of interest. Control and experimental conditions were tested in the same run. Means and SEMs were calculated from a minimum of 3 independent experiments.

### Primer sequences

JEP1966 ggggacagctttcttgtacaaagtggtaatggagctcgatcacactcca

JEP1967 ggggacaactttgtataataaagttgtttaataatggatgacgctttgac

JEP1974 ggggacaactttgtatagaaaagttgaaaagtgagcgacggattcc

JEP1975 ggggactgcttttttgtacaaacttgtctgatttttcacagtataattag

JEP1982 ggggacaactttgtatagaaaagttgtagagcatgtacctttatag

JEP1983 ggggactgcttttttgtacaaacttggggttttggtaggttttagt

## Supporting Information

Figure S1 **Alignment of the predicted NIPI-4 proteins.** Accession numbers for the different proteins are the following: *C. elegans* NP_505028, *C. remanei* XP_003115465, *C. brenneri* EGT43601, *C. briggsae* CAP37545, *C. japonica* JA58647. The *Ascaris* and *Pristonchius* proteins present in Genbank (ADY47863, PP41334) appear to have been mis-predicted. The figure presents more plausible predictions based on manual editing, respecting splice consensus sequences, of the output from tblastn using the *C. elegans* NIPI-4 protein against the relevant genomic sequence. All included sequences were found as significant matches with a smallest sum probability of at least e-25. For *Meloidogyne hapla*, *Oncocera volvulus*, *Strongyloides ratti* only partial sequences are presented, no attempt to reconstruct complete sequences was made (*). Alignments were produced with Clustal W2 (http://www.ebi.ac.uk/Tools/msa/clustalw2/) and Boxshade (http://www.ch.embnet.org/software/BOX_form.html). Thanks to G. Manning for the annotation of the different domains.
(DOC)

Figure S2 **Intestinal expression of NIPI-4 does not rescue the Nipi phenotype.** (A–C) wild type (A), *nipi-4(fr71)* (B) and *nipi-4*(*fr71*);P*vha-6*::NIPI-4 (C) worm strains carrying *frIs7* following infection. The expression of *nipi-4* in the intestinal cells in the adult does not rescue the *nipi-4* phenotype. Green and red fluorescence is visualized simultaneously. The green fluorescence at the level of the head and vulva in C is due to the co injection marker P*unc-53*::GFP [47].
(EPS)
