# Supplementary material for: The Pseudokinase NIPI-4 Is a Novel Regulator of Antimicrobial Peptide Gene Expression
Source: PLoS One. 2012 Mar 21;7(3):e33887. doi: 10.1371/journal.pone.0033887 (PMC3309975; doi:10.1371/journal.pone.0033887)
Supplement: Figure S1 — Alignment of the predicted NIPI-4 proteins. Accession numbers for the different proteins are the following: C. elegans NP_505028, C. remanei XP_003115465, C. brenneri EGT43601, C. briggsae CAP37545, C. japonica JA58647. The Ascaris and Pristonchius proteins present in Genbank (ADY47863, PP41334) appear to have been mis-predicted. The figure presents more plausible predictions based on manual editing, respecting splice consensus sequences, of the output from tblastn using the C. elegans NIPI-4 protein against the relevant genomic sequence. All included sequences were found as significant matches with a smallest sum probability of at least e-25. For Meloidogyne hapla, Oncocera volvulus, Strongyloides ratti only partial sequences are presented, no attempt to reconstruct complete sequences was made (*). Alignments were produced with Clustal W2 (http://www.ebi.ac.uk/Tools/msa/clustalw2/) and Boxshade (http://www.ch.embnet.org/software/BOX_form.html). Thanks to G. Manning for the annotation of the different domains. (DOC) [file pone.0033887.s001.doc]

# Figure S1

*C_remanei*  1 ----MDHTQPP--SVLNDNCSASYMTPYATVIAMSGLYLIVIYFFCKKSKKMCQPMSDSMYPYQKRLKQLERELKNFLID
*C_brenneri*  1 ----MDHSPPPE-SVLNDNCSASYMTPYATVIAMSGLYLIVIFFFCKKSKKMCQPMPDSMYPYQKRLKQLERELKNFLID
*C_briggsae*  1 ----MESTPPPPPSVLNDNCSASYMTPYATVIAMSGLYLIVIFFFCKKSKKMCIPMSDSMYPHQKRLKQLERELKNYLID
*C_elegans*  1 --MELDHTPPP--SVLNDNCSASYMTPYATVIAMSGLYLLAIFYFCKKSKKMCQPMSDSIYPYQKRLTQLERELKNYLID
*C_japonica*  1 MDHSSDHTEPPP-NLLDDNCSASYMTPYATVIAMSGLYLLAIFYFCKKSKKMCQPMSDSMYPYQKRIKQLERELKNYLID
*A_suum*  1 -----MQNESTS----AECTSTSYITPWATVIAMFSFYLLALFWFCKHGKSICLPAPDSIYPYKKRLKFLKRELAHLLVD
*M_hapla**  1 ------------------------------------------------GKNVCHPTPDSIFPYIERLRFLRR--------
*O_volvulus** 1 -----------------------------------------IALFFLDGKNLCIPAPDSLYPYQKRLKFLKR-LQHLLVD
*P_pacificus* 1 ------MTSPAQ----TTTCSAAYMTPWFTVAVMFALYILILLWFCKNGKKICMPTPDNIWPYEKRLKQLKRELSHLLLE
*S_ratti**  1 --------------------------------------IIKFKKKNLDRKSMCLPVPETIFPYTKRLKKLQKILEPYLVD
 //////////////////// *
 TM domain *fr106* nonsense
*C_remanei*  75 EESIEVDDYQIGQTADGFIFRGGVFPKTRNRFNAKVTTAVK-ISFPIVTKSISLLEDALRLSKLDHPNLIRLLAVSQLSF
*C_brenneri*  76 EESIEVDDYQIGQTADGFIFRGGVFPKTRNRFNAKVTTAVK-ISFPIVTKSISLLEDALRLSKLDHPNLIRLLAVSQLSF
*C_briggsae*  77 EESIEVDDYQIGQTSDGFIFRGGVFPKTRNRFNAKVTTAVK-ISFPIVGKSISLLEDALRLSKLDHPNLIRLLAVSQLSF
*C_elegans*  77 EESIEVDDFQIGQTADGFIFRGGVFPKTRNRFNAKVTTAVK-ISFPIVSKSISLLEDALRLSKLDHPNLIRLLAVSQLSF
*C_japonica*  80 EESIEVDDYQIGQTADGFVFRGGVFPKTRNRFNAKVTTAVK-ISFPIVTKSISLLEDALRLSKLDHPNLIRLLGVSQLSF
*A_suum*  72 DMFIELTDSKLGQGAVGFVFKGYVFPRTQTRFKQKVFAAVK-MSYPMPQKSMGLLEEAYRMSKLNHPHIVKLIAVSKLSF
*M_hapla**  25 ---IELTDIKLGQGAVGFVFKG--------------------MSYPMPQKSIGLLEEAARMARLNHPNIVKLIAVSKLSF
*O_volvulus** 39 DIFIELTDNKLGQGAVGFVFKGYVYPRTRSKLQRVFAAVKVNFSYPMPQKSIGLLEEAYRMSKLDHPNIVKLIAVSKLSF
*P_pacificus* 71 DMFIELCDHKLGQGSVGFVFKGFVYPNTQNRYKHKTPAAVK-MSYPLPAKSMGLLEEAARLARLSHPNIVKLLAVSQLSF
*S_ratti**  43 DVFIELTDHKLGQGSIGFVFKGFVYPKTQTRFKQKKCAAIK-LSYPIPQQSIGLLEEAARLSRLNHPNIVKLIAISRLSF
 ----------------------------------------------------------------------

*GQ***GF* K

Nucleotide phosphate-BD ATP-BD
*C_remanei*  154 TVFRPMIALEWLPGGTLADYFIY--KIREKEDSDRSPIQLKDMLSILYQVSQALKYIHSRLDEFGQELTHGRILTRNVLI
*C_brenneri*  155 TVFRPMIALEWLPGGTLSDYFIY--KIREKEDSDRSPIQLKDMLSILYQVSQALKYIHSRLDEFGQELTHGRILTRNVLI
*C_briggsae*  156 SVFRPMIALEWLPGGTLADYFIY--KIREKEDSDRSPIQLKDMLSILYQVSQALKYIHSQLDEFGQELTHGRILTRNVLI
*C_elegans*  156 TVFRPMIALEWLPGGTLADYFQF--KVREKDDSERSPIQLKDMLSILYQVSQALKYIHSQLDEFGQELTHGRIFTRNVLV
*C_japonica*  159 TVFRPMIALEWLPGGTLADYFLY--NVREKEDSERSPIQLKDMLSILYQVSQALKYIHCKLDEFGQELTHGRILTRNVLI
*A_suum*  151 QAFRPMIAMEWLPGGSLAEYFR--EHIRKRGN-DAPPVYVRDIVDILRQVGEALKYLHESRDAAGNEISHGDIAARNVLL
*M_hapla**  82 TAFRPMLALEWLPGGSLAEFFR--EEIRRKDERDQVRVYVRDIVHILIQIVHALKYLHENRDIQGNDFTHGDVAARNILL
*O_volvulus** 119 QAFRPMIAIEWLPGGSLLEYFR----------------------------------------------------------
*P_pacificus* 150 SALRPMLVIEWLPGGSLGEYFR--LNCRNPND--DPVVYVRDVVRLLQQVAAALQKDLQKRDADGSELTHRDVAARNVLL
*S_ratti**  122 SAFRPMFAMEWLSGGSLAEFFKNLRTSKNENEKYGKKVYVRQIINVLSQVGEALKYIHESRDQDGSEFTHGDVAARNVLL
 -----------------------------------------------------------------------$--------
 Protein Kinase domain Active site (D)

*C_remanei*  232 SEPDLKKCEVKLGDFGEAPSGLEYATPIVAYMPPEILCCAEK-----IPPHRPENDVWMFGVFIWECLTLGAQPHFRKSV
*C_brenneri*  233 SEPDLKKCEVKLGDFGEAPSGLEYSTPIVAYMPPEILCCAER-----IPPHRPENDVWMFGVFIWECLTLGAQPHFRKSV
*C_briggsae*  234 TEPDLKKCEVKLGDFGEAPSGLEYTTPIVAYMPPEILCCAER-----IPPHRPENDVWMFGVFIWECLTLGAQPHFRKSV
*C_elegans*  234 TEPDLRKCEVKLGDFGDAPMGLEYSTPIIAYMPPEILCCAER-----IPPHRPENDVWMFGVFIWECLTLGAQPHFRKSV
*C_japonica*  237 SEPDLKKCEVKLGDFGEAPMGLEYVTPIVSYMPPEILCCAER-----IPPHRPENDVWMFGVFIWECLTLGAQPHFRKSV
*A_suum*  228 TSTKLQRCKAKLGDFG-LPLDFGPRLPIP-WLPPEIVSCLD----RTRIQHRPESDVWMFGVLGWECATLGAEPHYQRTV
*M_hapla**  160 NERNLATCIAKLGDFG-LPIDFGPRLPIP-WLPPEIVCSLD----QQNTKHRPESDVWMFGVLCWECATLGAEPT-----
*P_pacificus* 226 TSTDLTKCNAKLGDFG-LPIDFGNHLPLP-WLPPEIVGCATSHDPKARTRHRPEADVWMFGVLSWECATLGAEPHYQRSV
*S_ratti**  202 THRDLENCIAKLGDFG-LPIDFGPRLPIP-YLPPEIVCAAD----FLQCVHRVEADVYMFGVLGWECATLGAEPHYQKSF
 ----------------------------------------------------------------------------------
 +++++++++++*++++++ * *

Activation loop (* Y phosphorylation) *fr68* *fr71*

G->E nonsense
*C_remanei*  307 EDIKKSFRLPDRGLSCPPTCPLDVWTLVIDCLSDPHVRPRFASTTN--ASITMRLSELHHIVSPALFLYPIPNQS-VCTC
*C_brenneri*  308 EDIKKSFRLPDRGLSCPPTCPLDVWTLVIDCLSDPHVRPRFASTTN--ASITTRLSELHHIVSPALFLYPIPNQS-VCTC
*C_briggsae*  309 EDIKKSFRLPDRGLSCPPTCPLDVWTLVIDCLSDPHVRPRFASTTN--ASITTRLSELHHIVSPALFLYPIPNQS-VCTC
*C_elegans*  309 EEIKKSFRLPDRGLSCPPTCPLDVWTLVSDCLSEPHMRPRFASTTN--ASITSRLSELHHIVSPALFLYAIPNQS-VCTC
*C_japonica*  312 DEIKKSFRLPDRGLSCPPTCPLDVWTLVSDCLSEPHIRPRFASTTN--ASIPSRLSDLHHIVSPALFLYPIPNQS-VCTC
*A_suum*  301 DEIQQCFTWPDRGLHRPPSCPLDFWNFLLDCMSEQHRRPRFAGPTDTVSSAIYRLRVLQLMYERNDHTFEIYYNTSNCTC
*M_hapla**  229 -------------------------DFVYDCLSEQHRRPRFAGPTDVYSSAIFRLTHIQELYKHSRARFRMVQNVSNCTC
*P_pacificus* 304 REINTTPFQ-DRGLPCPPNCPAIFFDFVQSCLSEPHRRPSFVAPIDSSSSVLFRCFDLWSL---------MFDLCSSCTC
*S_ratti**  276 EEIQKSLSERDRGLKRPKHTPDD---------------------------------------------------------
 -----------------

*C_remanei*  384 IEHHCQSIPQY----
*C_brenneri*  385 IEHHCQSVPQY----
*C_briggsae*  386 IEHHCQSIPQY----
*C_elegans*  386 IEHHCQSVIHY----
*C_japonica*  389 TEHHCRSVPQY----
*A_suum*  381 HQHRCNVPLPSFSGR
*M_hapla**  284 AQHRC----------
*P_pacificus* 374 ATHRCARPRM-----
